# Identifying trajectories and predictors of chemotherapy-induced peripheral neuropathy symptoms, physical functioning, and falls across treatment and recovery in adults treated with neurotoxic chemotherapy: the PATTERN observational study protocol (NCT05790538)

**DOI:** 10.1186/s12885-023-11546-2

**Published:** 2023-11-10

**Authors:** Kerri M. Winters-Stone, Stephanie M. Krasnow, Fay B. Horak, Martina Mancini, Michelle H. Cameron, Nathan F. Dieckmann, Sydnee A. Stoyles, Eric J. Roeland

**Affiliations:** 1 Knight Cancer Institute, School of Medicine, Oregon Health & Science University, 3181 SW Sam Jackson Park Road, Portland, OR 97239 USA; 2 Department of Neurology, School of Medicine, Oregon Health & Science University, Portland, OR USA; 3 VA Portland Health Care System, Portland, OR USA; 4 School of Nursing, Oregon Health & Science University, Portland, OR USA; 5 Division of Psychology, Department of Psychiatry, School of Medicine, Oregon Health & Science University, Portland, OR USA

**Keywords:** Cancer, Neuropathy, Mobility, Balance, Gait, Paresthesia, Pain, Neurotoxicity, Side effects

## Abstract

**Background:**

Chemotherapy-induced peripheral neuropathy (CIPN) is a debilitating and dose-limiting side effect of systemic cancer therapy. In many cancer survivors, CIPN persists after treatment ends and is associated with functional impairments, abnormal gait patterns, falls, and diminished quality of life. However, little is known regarding which patients are most likely to develop CIPN symptoms that impair mobility and increase fall risk, when this risk develops, or the optimal timing of early intervention efforts to mitigate the impact of CIPN on functioning and fall risk. This study will address these knowledge gaps by (1) characterizing trajectories of symptoms, functioning, and falls before, during, and after treatment in adults prescribed neurotoxic chemotherapy for cancer; and (2) determining the simplest set of predictors for identifying individuals at risk for CIPN-related functional decline and falls.

**Methods:**

We will enroll 200 participants into a prospective, observational study before initiating chemotherapy and up to 1 year after completing chemotherapy. Eligible participants are aged 40–85 years, diagnosed with stage I-III cancer, and scheduled to receive neurotoxic chemotherapy. We perform objective assessments of vibratory and touch sensation (biothesiometry, tuning fork, monofilament tests), standing and dynamic balance (quiet stance, Timed-Up-and-Go tests), and upper and lower extremity strength (handgrip dynamometry, 5-time repeated chair stand test) in the clinic at baseline, every 4–6 weeks during chemotherapy, and quarterly for 1 year post-chemotherapy. Participants wear devices that passively and continuously measure daily gait quality and physical activity for 1 week after each objective assessment and self-report symptoms (CIPN, insomnia, fatigue, dizziness, pain, cognition, anxiety, and depressive symptoms) and falls via weekly electronic surveys. We will use structural equation modeling, including growth mixture modeling, to examine patterns in trajectories of changes in symptoms, functioning, and falls associated with neurotoxic chemotherapy and then search for distinct risk profiles for CIPN.

**Discussion:**

Identifying simple, early predictors of functional decline and fall risk in adults with cancer receiving neurotoxic chemotherapy will help identify individuals who would benefit from early and targeted interventions to prevent CIPN-related falls and disability.

**Trial registration:**

This study was retrospectively registered with ClinicalTrials.gov (NCT05790538) on 3/30/2023.

## Introduction

Chemotherapy-induced peripheral neuropathy (CIPN) is a persistent cancer treatment-related side effect that negatively impacts physical functioning, falls, and quality of life [1–4]. CIPN typically affects the hands and/or feet, with numbness, tingling, pain, cold hypersensitivity, and muscle weakness among the most common sensory and motor symptoms [5, 6]. CIPN is estimated to occur in 25%-90% of patients during chemotherapy and persists in many survivors long-term [7–9]. Multiple classes of neurotoxic chemotherapy drugs are associated with CIPN, including taxanes, platinum derivatives, and vinca alkaloids [10]. The etiology and natural history of CIPN are heterogeneous across classes of neurotoxic chemotherapeutic agents [11, 12]. Not all patients receiving the same neurotoxic treatment regimen will develop CIPN, but for those who do, symptom severity and recovery after treatment vary widely [13].

Cancer survivors with CIPN have worse physical functioning (e.g., mobility), more self-reported disability, and significantly higher fall rates than asymptomatic survivors [4, 7, 14]. In a cross-sectional study of breast cancer survivors approximately 6 years post diagnosis, distinct patterns of worsening objective and patient-reported physical function, disability, and fall risk were associated with increasing CIPN severity [4]. Most studies reporting mobility impairments due to CIPN have multiple limitations: cross-sectional design, reliance on complex measures that are impractical to capture in a clinic setting, and lack of power to capture falls. Moreover, these studies do not examine heterogeneity across patients, which may be central to identifying patients at greatest risk for poor outcomes [4, 15, 16]. Longitudinal studies reporting trajectories of CIPN symptoms and mobility deficits have been conducted, but none have linked mobility impairments to falls or the onset of disability [17, 18]. Additionally, it remains unknown when CIPN symptoms become severe enough to impair functioning and increase fall risk.

Until a simple system for identifying when and which patients are at risk for CIPN-related mobility deficits can be incorporated into the clinical workflow and used to target early intervention strategies, falls and disability from CIPN will likely remain underrecognized and undertreated. A critical first step toward identifying simple, early predictors of functional decline and developing fall risk is to characterize the natural trajectories of symptoms, functioning, and falls across the in-treatment and recovery phases of cancer care. Thus, we are conducting a longitudinal observational study examining patterns of CIPN symptom development, disability onset, and falls in adults with cancer before, during, and after neurotoxic chemotherapy. We simultaneously track symptoms, functioning, and falls through patient-reported symptoms, disability, and physical functioning; objective physical performance measures; and continuous passive monitoring of physical activity and mobility during chemotherapy and 1 year into recovery.

## Methods

### Study aims and hypotheses

Our study aims and hypotheses are three-fold: (1) Characterize the variability in trajectories of CIPN symptoms and physical functioning in patients across treatment and 1 year of recovery. We hypothesize that there will be significant congruence between worsening CIPN symptoms and declining physical activity, mobility, and self-reported physical functioning and disability; (2) Identify 2 or more distinct trajectories of change in symptoms and functioning associated with significant differences in fall rates. We hypothesize that we can identify 2 or more distinct trajectories of CIPN and physical functioning associated with falls; and (3) Identify the simplest set of predictors that identify patients at risk for CIPN-related functional decline and falls. We hypothesize that some combination of patient characteristics (e.g., age, sex, comorbidities, body mass index), chemotherapy features (e.g., chemotherapy drug class, number of chemotherapy cycles, cumulative chemotherapy dose), and mobility measures (gait, balance) will predict distinct trajectories of change in CIPN symptoms and physical functioning [9, 19, 20].

### Study design and setting

This single-center, prospective, observational study is being conducted at the Knight Cancer Institute at Oregon Health & Science University (OHSU). Participants are recruited from the main OHSU Knight Cancer Institute clinic and 5 OHSU Knight Cancer Institute community hematology-oncology clinics around the Portland metropolitan area. The study was approved by the OHSU Institutional Review Board (#21969) and registered with ClinicalTrials.gov (NCT05790538).

### Study participants

The target population is adults with cancer scheduled to receive neurotoxic chemotherapy with curative intent. To be eligible to participate, participants must meet the following eligibility criteria:

1. Age 40–85 years on the date of enrollment. We chose this age range because the side effects and symptoms from chemotherapy may differ between young, middle-aged, and advanced-aged patients, and the subsequent impact on mobility may be similarly diverse. We exclude adolescent and young adult patients (15–39 years old) who may be less vulnerable to falls and disability at a young age and adults over 85 years to limit mobility/falls associated with advanced age and multi-morbidities. The age range of patients in the study is broad enough to examine the influence of age on study outcomes.
2. Diagnosed with stage I-III cancer, or stage IV cancer considered curable, other than cancers or metastases in the brain or spinal cord.
3. Patients scheduled to receive neurotoxic chemotherapy, including taxane derivatives, platinum complexes, and/or vinca alkaloids.
4. Patients without prior receipt of neurotoxic chemotherapy.
5. Patients without cognitive difficulties that preclude answering survey questions, participating in performance tests, or giving informed consent as determined by the referring provider.
6. Patients must be free of a medical condition, movement or neurological disorder, or medication use that contraindicates participation in mobility testing and/or confounds the ability to detect treatment-related changes in balance and mobility. Specific medical conditions include, but are not limited to, severe muscular dystrophy, severe spasticity, epilepsy, seizures, Alzheimer’s disease, and dementia; specific physical conditions include a severe balance disorder (e.g., from late-stage Parkinson’s disease or stroke), inability to ambulate (use of an assistive device is permitted), inability to stand for 3 min, or severe hearing or vision problems.

### Participant recruitment and retention

We plan to enroll 200 participants. Patients are identified through chart reviews, tumor boards, and direct referrals from oncology clinicians. With the clinician’s permission, a research coordinator contacts the patient by phone to explain the study and screen for eligibility. Written informed consent is obtained electronically via the OHSU electronic data management system (REDCap) before or at the first study visit. For patients we cannot contact prior to their first chemotherapy infusion, or who express that they are too overwhelmed for a research visit at their first infusion, we offer to delay study enrollment until the second chemotherapy cycle. Patients are not enrolled after their second chemotherapy cycle because important information about early changes in symptoms and function can no longer be obtained.

We use several strategies to retain participants in the study. One research assistant is assigned to each participant throughout the study to establish a trusting relationship between each participant and a research team member. We minimize participant burden by using home-based data collection (online surveys and passive monitoring devices) and by conducting testing visits in the clinic just prior to regularly scheduled oncology appointments. If participants do not have a scheduled post-treatment clinic visit within the desired time frame, they are asked to schedule a separate research visit at the Knight Cancer Institute. Participants are remunerated for each study visit ($25 for research visits that coincide with clinic visits or $50 for separate post-treatment research visits). Travel compensation is provided for participants who live > 30 miles from OHSU to attend separate post-treatment research visits.

### Procedures

**Fig. 1:**
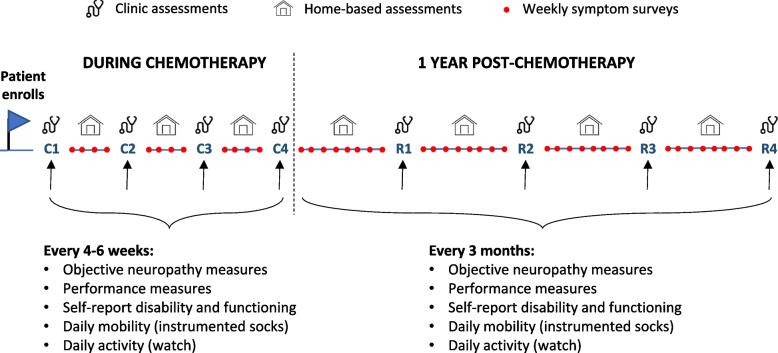
Data collection timeline. Data are collected in the clinic and at home. Participants complete objective neuropathy and performance assessments at chemotherapy infusion visits (C1, C2, C3…) every 4–6 weeks and at follow up oncology visits (R1-R4) every 3 months during the first year of recovery. At home, participants wear wearable devices for 4–7 days following each clinic assessment and complete weekly electronic surveys throughout the study

Data collection uses the following methods: (a) clinic-based assessments of vibration and tactile sensation for CIPN and performance tests of physical functioning, (b) home-based assessments of symptoms and functioning using web-based surveys and wearable devices to monitor daily physical activity and mobility, and (c) data on cancer history, medications, and chemotherapy regimens abstracted from the electronic health record (EHR) (Fig. 1).

Clinic-based data collection occurs during chemotherapy according to each patient’s treatment schedule and post-chemotherapy at quarterly intervals for 1 year. The baseline clinic assessment occurs before the first or second chemotherapy cycle, and then assessments are repeated every 4–6 weeks (coincident with the treatment schedule) until the final chemotherapy infusion. CIPN and performance measures are taken during clinic visits before chemotherapy administration. Post-chemotherapy (i.e., recovery), clinic assessments are performed with routine follow-up care at approximately 3-, 6-, 9-, and 12-months post-chemotherapy.

Home-based data collection consists of web-based surveys and wearable devices. Survey invitations are sent via SMS or email according to individual preference. Brief symptom and fall surveys are administered weekly during the study. More detailed physical functioning and disability surveys are administered at time points coinciding with clinic assessments. At the end of each clinic assessment, participants receive wearable devices that measure physical activity and gait quality passively and unobtrusively in their homes. Participants are asked to wear the devices for at least 4 of the next 7 days and at least 6–8 h per day during waking hours. After 7 days, participants mail the devices back to OHSU in pre-paid, pre-addressed shipping boxes or return them to study staff at their next clinic assessment.

### Outcome measures

#### Symptoms

##### Objective CIPN measures

Loss of perceived sensation is a widely accepted clinical measure to screen for CIPN [21]. We perform 3 tests of loss of perceived sensation.

1. *Tuning fork test*: Vibratory sensation is measured by placing a 128 Hz vibrating tuning fork (US Neurologicals, LLC, Poulsbo, WA) proximal to the nail bed on the right dorsal hallux and recording the time that elapses before the participant indicates they can no longer detect vibration [22]. Three trials of the test are conducted and then averaged.
2. *Monofilament test*: The tester records the number of times an individual detects 4 brief applications (~ 1 s) of a 10-g monofilament (Medical Monofilament, Plymouth, MA) on the sole of each foot, for a total of 8 sites [22].
3. *Biothesiometry*: The lowest amplitude of vibration that an individual can detect, or vibration perception threshold (VPT), is assessed using a biothesiometer (Biothesiometer USA, Avon Lake, OH) [23]. A handheld device that varies vibration amplitude at a constant frequency is placed proximal to the nail bed on the right dorsal hallux. The VPT is measured by slowly increasing the amplitude from 0 V until the individual indicates that they detect vibration in their toe. Two trials are performed and averaged to generate the VPT. Biothesiometry is only performed at the final chemotherapy infusion and at all post-chemotherapy assessments.

##### **Patient-**reported** CIPN**

Patient-reported CIPN is assessed using the Patient-Reported Outcomes version of the Common Terminology Criteria for Adverse Events v1.0 (PRO-CTCAE™: https://healthcaredelivery.cancer.gov/pro-ctcae/) reporting tool and the Functional Assessment of Cancer Therapy – Gynecologic Oncology Group Neurotoxicity 13-item questionnaire (FACT-GOG-Ntx-13) [24–27]. Two PRO-CTCAE™ items are administered weekly, asking the patient to report whether they experienced numbness or tingling in the hands or feet and how much the symptom interfered in their daily life during the last 7 days. We also administer the 13-item neuropathy subscale of the FACT-GOG-Ntx-13 which assesses the presence, severity, and interference of neuropathy symptoms in patients receiving neurotoxic chemotherapy. The scale ranges from 0–52, with lower scores indicating worse quality of life due to neuropathy. Both surveys are sent weekly for the duration of the study.

##### Other symptoms

Patient-reported symptoms (dizziness, cognitive function, pain, insomnia, fatigue, anxiety, and depressive symptoms) are assessed with PRO-CTCAE™ reporting tool items that assess the severity, frequency, and interference of each symptom in daily life during the previous 7 days [25]. Symptom surveys are sent weekly for the duration of the study.

### Physical functioning

#### Objective performance measures

##### Upper extremity strength

Upper extremity strength is measured via hand grip dynamometry, an approach used in standard neuropathy assessments to determine whether neuropathy involves motor neurons [21, 28, 29]. Grip strength in the dominant hand is measured with a handgrip dynamometer (Creative Health Products, Ann Arbor, MI). Participants stand with their feet shoulder-width apart and arms hanging loosely at their sides. Participants are first cued to inhale and then prompted to exhale while squeezing the dynamometer as hard as they can, and the maximum force (in kg) is recorded. The procedure is repeated 2 more times for a total of 3 trials, which are then averaged.

##### Lower extremity function

Lower extremity function is measured by a timed chair stand test, measured as seconds required to rise from a sitting position in a chair to a full stand 5 times in a row [30–32]. Chair stand times > 12 s have been shown to predict a 2.4-fold increased risk of falls in older adults [33].

##### Static balance

Static balance is assessed by a postural sway test that measures how well a person can maintain their balance during quiet standing. Increased sway indicates poor balance control, is associated with an increased risk of falls, and is greater in populations with neurologic impairment [34, 35]. Postural sway is measured with a Mobility Lab Opal sensor and software (APDM Wearable Technologies, Clario, Portland, OR). The wireless, Opal inertial sensor placed on the lumbar region captures multiple postural sway variables, including total sway area (m^2^/s^4^) and mean sway velocity (m/s). Participants are asked to stand as still as possible with feet together and hands on hips for 30 s. The test is first conducted with eyes open and then repeated with eyes closed.

##### Dynamic balance

Dynamic balance is measured by the Timed-Up-and-Go (TUG) test, a widely accepted clinical measure of dynamic balance in older adults and clinical populations [36]. The TUG test evaluates the time in seconds that it takes an individual to rise from a chair, walk 3 m at their usual pace, turn around, and return to a seated position in the chair. The procedure is repeated for a total of 2 averaged trials to calculate TUG time. Slower TUG times are associated with an increased risk of falls and disability and have been observed in taxane-treated breast cancer survivors relative to healthy controls [31, 37–39].

#### Daily mobility

Daily mobility is measured with instrumented socks with inertial sensors embedded into thin, neoprene fabric (APDM Wearable Technologies, Clario) [40–42]. For 4–7 days after each clinic assessment, participants are asked to wear the socks during waking hours for at least 6–8 h and then charge them overnight. Raw data are stored on the sensors and then uploaded to a secure server for analysis with Motion Studio software (APDM Wearable Technologies, Clario). Examples of mobility measures calculated during daily monitoring are reported in Table 1. Mobility measures are calculated with proprietary algorithms from APDM, many of which have been validated [43–45]. The measures are calculated by combining the 3 axes of linear acceleration with 3 axes of angular velocity and the magnetometer (all sampled at 128 Hz) to obtain the orientation of the limb in space. Wireless synchronization allows precise temporal binding of data across limbs. Walking bouts and turning events are first identified, followed by calculating metrics within each walking bout and turning event.

**Table 1:** Selected mobility measures obtained by the instrumented socks

Activity	Gait Quality	Turning Quality
% of day active	Stride velocity (cm/s)	# turns/hour
# walking steps/day	Stride length (m)	Turn velocity (°/s)
# walking bouts/day	Cadence (steps/min)	Turn angle (°)
Duration of longest bout/day (s)	Double support time (ms)	Turn duration (s)
	Angle of foot at heel strike (°)	Turn angle/step (°/step)

#### Daily physical activity

Daily physical activity is measured using accelerometry, which is considered the gold standard for objective physical activity monitoring and has been used during various phases of cancer care [46]. Participants are given an ActiGraph GT9X Link watch (Actigraph, Pensacola, FL). They are asked to wear it on their non-dominant wrist for 24 h per day for 4–7 days after each clinic assessment so that objective measures of sleep can be obtained and considered in future analyses. Participants who are unwilling to wear a watch while sleeping are asked to wear the watch for at least 6–8 h per day during waking hours for 4–7 days after each clinic assessment to obtain daily activity data. Data are analyzed with ActiLife software (version 6) [47]. Of particular interest in this study are total energy expenditure (kcal/d) and time spent in sedentary, light, and moderate-vigorous activities over 7 days after each clinic assessment.

#### Patient-reported physical functioning

Perceived physical function is measured using the physical functioning subscale of the European Organization for Research and Treatment of Cancer Quality of Life Questionnaire (EORTC QLQ-C30) [48]. The physical functioning subscale of the QLQ-C30 is valid and reliable [48, 49]. Scores on this subscale range from 0–100, with higher scores indicating better functioning. This survey is administered at time points corresponding to each clinic assessment.

#### Patient-reported disability

Perceived disability is evaluated using the disability component of the Late-Life Function and Disability Instrument (LLFDI), which assesses disability in terms of frequency and limitation in performing 16 life tasks [50]. The LLFDI is a valid and reliable instrument that we have previously used to document significantly more disability in cancer survivors with CIPN than in asymptomatic survivors [4, 50]. Scores range from 0 to 100, with higher scores indicating less disability. This survey is administered at time points corresponding to each clinic assessment.

#### Quality of life

Quality of life is assessed using the EORTC QLQ-C30 [48]. Global quality of life and subdomains (physical, emotional, social, role, and cognitive functioning) are scored from 0–100, with higher scores indicating better quality of life or functioning. The individual subscales and summary scores are valid and reliable [48, 49], and good test–retest reliability and acceptable validity have been demonstrated in patients with CIPN [22]. This survey is administered at time points corresponding to each clinic assessment.

### Falls

#### Falls

Falls that occurred during the 12 months prior to enrollment are assessed retrospectively at baseline, and falls that occur prospectively are assessed at weekly intervals using in-house surveys. These surveys ask patients about the number of falls, the nature of any injurious falls, and medical care resulting from a fall during the observation period. A fall is defined as unintentionally coming to rest on the ground or at some other lower level, not due to a major intrinsic event (e.g., stroke or syncope) or overwhelming hazard [51]. An injurious fall is one that results in fractures, head injuries, sprains, bruises, scrapes, or serious joint injuries, or where the participant seeks medical care [51].

### Descriptive variables and other measures of interest

Demographic variables, medications (including chemotherapy), cancer health history, and body mass index are collected at baseline and updated throughout the study via the EHR and an in-house questionnaire. The presence of chronic health conditions is measured with the Charlson Comorbidity Index [52]. Cumulative chemotherapy doses and relative dose intensities are calculated from the EHR.

### Statistical analysis

#### Overview of modeling strategy

We will employ a *modified* growth mixture modeling (GMM) approach. GMM identifies distinct patterns of change across time that vary around different means and have unique variance and homogenous within-trajectory growth. Cases are then assigned to the “most likely class” or pattern of change over time. Changes over time are modeled as random effects and non-normal distributions and non-linear patterns of change can be modeled [53]. Typically, the growth and latent class aspects of GMMs are estimated simultaneously within a structural equation modeling (SEM) framework, although these models are much easier to estimate with time-structured data (i.e., participants are measured the same number of times at the same intervals) [54]. In the present study, measurements will not be time structured, and we will employ a modified GMM approach. With this approach, the initial growth modeling will be done with a mixed-effects model (stage 1), which flexibly handles non-time structured data, and then we will submit the intercept and growth estimates from this model to a latent class (aka latent profile) model within the SEM framework (stage 2). All analyses will be performed using R v4.2.2 [55] and Mplus v8.7 [56].

#### Handling missing data

The mixed-effects growth models will be estimated with maximum likelihood, which utilizes all available outcome data for each participant without removing participants due to missingness [57]. For the GMM stage, full-information maximum likelihood (FIML) estimation will be used to handle data that is missing completely at random (MCAR) or missing at random (MAR). For data that are MAR, variables related to the missingness will be included using the auxiliary command in Mplus (i.e., to allow unbiased parameter estimation under MAR) while not including them in the data analysis model [58]. These approaches will allow the analysis of the proposed aims in the case of patient withdrawal, maximizing statistical precision and power.

#### Confounding

We will test the influence of and include, as appropriate, all known confounders in our longitudinal and latent class models.

#### Specific analysis plan

The analysis will proceed in a series of stages.

*Hypothesis 1.1:Worsening neuropathy symptoms will be associated with concomitant worsening in functioning.*

The change over time in each symptom and functioning outcome will be modeled using a mixed-effects modeling framework implemented in the lme4 statistical package for R. The fixed and random effects structure and the shape of change across time (e.g., linear versus nonlinear) will be determined through likelihood ratio model testing and information criteria. Intercepts (initial status) and growth estimates (e.g., linear and/or quadratic trends) will be estimated for each individual patient for each outcome. All pairs of neuropathy symptom and functioning intercepts and slopes will be correlated to test Hypothesis 1.1*.*

*Hypothesis 1.2. At least two classes of patients with distinct trajectories of change in symptoms and functioning can be identified, and will be associated with significant differences in fall rates.*

 Each patient's intercept and growth estimates for each measure will be entered into a GMM to identify patient clusters with distinct symptom and function trajectory profiles. For each patient, a probability of belonging to each latent class will be assigned, and the highest membership probability will be used to make a final class assignment. Identification of the best-fitting model will be based on information criteria (e.g., Bayesian Information Criterion), where smaller numbers indicate a better fit, convergence (entropy > 0.80), the proportion of the sample in each latent class (not < 5%), posterior probabilities (mean probability of belonging in “most likely” class near 1.0), and the Lo-Mendell-Rubin adjusted likelihood ratio test (which is used to compare alternative models, e.g., k vs. k-1 classes) [53]. After a good fitting GMM model is found, the resulting latent classes will be described with respect to differences between initial CIPN symptom and functioning profile and change across time. Standard statistics (e.g., t-tests) will then be used to compare fall rates between the observed symptom and function latent classes. We will focus interpretation on effect sizes and 95% CIs. Beyond examining fall rates across the study time course, we will also explore survival models to predict time to first fall. Kaplan Meier models, Mantel-Cox log-rank tests and generalized Wilcoxon-Breslow tests will be used to evaluate differences in the distribution of time to first fall. Multivariate hazard ratios with 95% CIs and Wald χ2 statistics will be calculated from Cox models with Schoenfeld residuals used to test proportional hazards assumptions.

*Hypothesis 2.1. Sociodemographic (e.g., age, sex, comorbidities, body mass index), clinical (e.g., type of chemotherapy, # of chemotherapy cycles and/or cumulative dose of chemotherapy, disease stage, other symptoms), and mobility measures (gait and balance) can be used to predict distinct trajectories of change in neuropathy symptoms and functioning.*

 Standard statistical tests will be used to examine the association between latent class membership and sociodemographic, clinical, and mobility measures depending on level of scale (e.g., Kendall/s tau-b or Kruskal–Wallis tests). Since we will recruit both men and women into the study, we can also address sex as a biologic variable in the context of symptoms, functioning, and falls among cancer survivors**.** Thus, we will be the first to determine if sex associates with specific symptom trajectories that might lead to either gender-specific management approaches or a uniform strategy. Since there is a potentially large number of variables that will be compared we will control for false discovery rates with the Hochberg adjustment [59].

### Sample size/power calculation

Although simulation methods are available to help estimate sample size for

GMM, this requires known values for all model parameters and is not defensible given the unknowns in this domain. With 2 symptom measures (severity and interference) and 6 function measures in our most complex GMM model, however, our n-to-items ratio meets conservative sample size recommendations for related approaches (20:1) [60] and would require 160 complete cases. To maximize statistical power we will fit parsimonious models after extensive preliminary associative analyses. Regarding class comparisons, assuming 80% power, two-sided α of 0.05, and 2 equal groups, we will be able to detect small-medium sized differences in fall rates and other continuous variables between groups (Cohen’s *d* = 0.40). For categorical variables, we will be able to detect small-medium effects (w = 0.25) for cross-tabulation tables with as many as 4 degrees of freedom. Evaluable data on 200 participants is a feasible number and will allow us to address each aim while accounting for up to ~ 20% attrition.

## Discussion

CIPN is a distressing and disabling side effect of systemic cancer therapy. In addition, severe CIPN during treatment often results in dose reductions or discontinuation of chemotherapy, which may reduce therapeutic efficacy and worsen prognosis. After cancer treatment ends, lingering CIPN continues to negatively impact physical functioning and quality of life in many survivors. The incidence of CIPN is rising because more neurotoxic agents have been developed and are used to treat a wider variety of cancers, multi-drug chemotherapy regimens have become more common, and cancer survivors are living longer [10]. Currently, clinical guidelines for management of CIPN focus on pain relief, leaving clinical practice with little information about in whom, when, and how to intervene to prevent other downstream consequences of CIPN [10]. The present study will address these knowledge gaps by identifying risk profiles associated with CIPN-related mobility impairments, points in care where early intervention might be best timed to prevent disability and falls, and which mobility deficits to target.

To our knowledge, this is the first longitudinal study to simultaneously track objective and patient-reported CIPN, physical functioning, mobility, physical activity, and falls across chemotherapy and into recovery. In addition to performance measures obtained in the clinic setting, we use wearable devices to measure the quantity of activity and quality of mobility in patients’ everyday living environments. Measures of mobility during daily life are more sensitive than clinical tests at detecting impairments leading to fall risk in older adults [45]. In the present study, the use of wearable devices to capture early changes in functional mobility and activity across treatment and recovery may improve the ability to detect impending functional decline and future fall risk, even before decrements are observed with standard tests of gait and balance or patients report mobility problems.

Once we characterize the variability in symptom and functioning trajectories, we will determine the simplest set of predictors that identify patients at risk for CIPN-associated functional decline and falls. We will consider multiple patient, behavioral, and clinical attributes that may increase predictive capacity for CIPN. Several possible risk factors that increase the likelihood of developing CIPN have been suggested, including comorbidities, age, smoking, obesity, and inactivity [9, 19, 20]. Cross-sectional studies have identified single attributes associated with higher CIPN, but only a longitudinal study can differentiate between causal factors and co-occurring problems to identify which and why patients are at risk for CIPN, associated mobility problems, and/or falls. We will also evaluate whether treatment characteristics (e.g., number of cycles, cumulative dose, etc.) and simple mobility tests (e.g., TUG) that can quickly be performed in a clinic setting improve predictive power.

Determining how, when, and in whom neurotoxic cancer treatment increases the risk of falls and disability will provide a more nuanced and comprehensive understanding of the presentation and progression of treatment-related impairments. Early identification of simple, early predictors of functional decline and developing fall risk would enable providers to closely monitor those patients at greatest risk of developing CIPN that becomes severe enough to limit mobility and increase fall risk. This knowledge would also facilitate shared decision-making between providers and patients regarding balancing clinical outcomes and quality of life during treatment planning. Incorporating simple measures of impending functional decline into clinical pathways would alert providers when symptoms progress enough to warrant interventions such as dose reductions, switching treatments, and/or referral to rehabilitation. Identifying which mobility deficits to target would contribute to an evidence base for preventive and rehabilitative programs that improve function and in turn lower fall and disability risk. Collectively, these practices would mitigate disability and falls associated with cancer treatment and improve quality of life in cancer survivors.

## Data Availability

The datasets used during the current study are available from the corresponding author on reasonable request.

## Abbreviations

CIPN: Chemotherapy-induced peripheral neuropathy
EHR: Electronic health record
EORTC QLQ-C30: European Organization for Research and Treatment of Cancer Quality of Life Questionnaire
FACT-GOG-NTX-13: Functional Assessment of Cancer Therapy/Gynecologic Oncology Group Neurotoxicity 13-item Questionnaire
FIML: Full-information maximum likelihood
GMM: Growth mixture modeling
LLFDI: Late-Life Function and Disability Instrument
MAR: Missing at random
MCAR: Missing completely at random
OHSU: Oregon Health & Science University
PRO-CTCAE: Patient-Reported Outcomes version of the Common Terminology Criteria for Adverse Events
REDCap: Research Electronic Data Capture
SEM: Structural equation modeling
TUG: Timed-Up-and-Go
VPT: Vibration perception threshold
